# Multi-modal self-adaptation during object recognition in an artificial cognitive system

**DOI:** 10.1038/s41598-022-07424-9

**Published:** 2022-03-08

**Authors:** David Miralles, Guillem Garrofé, Carlota Parés, Alejandro González, Gerard Serra, Alberto Soto, Xavier Sevillano, Hans Op de Beeck, Haemy Lee Masson

**Affiliations:** 1 GTM - Grup de Recerca en Tecnologies Mèdia, La Salle-Universitat Ramon Llull, Barcelona, Catalonia Spain; 2 Department of Brain and Cognition, Leuven Brain Institute, KU Leuven, Leuven, Belgium; 3 Department of Cognitive Science, Johns Hopkins University, Baltimore, MD USA

**Keywords:** Computer science, Perception

## Abstract

The cognitive connection between the senses of touch and vision is probably the best-known case of multimodality. Recent discoveries suggest that the mapping between both senses is learned rather than innate. This evidence opens the door to a dynamic multimodality that allows individuals to adaptively develop within their environment. By mimicking this aspect of human learning, we propose a new multimodal mechanism that allows artificial cognitive systems (ACS) to quickly adapt to unforeseen perceptual anomalies generated by the environment or by the system itself. In this context, visual recognition systems have advanced remarkably in recent years thanks to the creation of large-scale datasets together with the advent of deep learning algorithms. However, this has not been the case for the haptic modality, where the lack of two-handed dexterous datasets has limited the ability of learning systems to process the tactile information of human object exploration. This data imbalance hinders the creation of synchronized datasets that would enable the development of multimodality in ACS during object exploration. In this work, we use a multimodal dataset recently generated from tactile sensors placed on a collection of objects that capture haptic data from human manipulation, together with the corresponding visual counterpart. Using this data, we create a multimodal learning transfer mechanism capable of both detecting sudden and permanent anomalies in the visual channel and maintaining visual object recognition performance by retraining the visual mode for a few minutes using haptic information. Our proposal for perceptual awareness and self-adaptation is of noteworthy relevance as can be applied by any system that satisfies two very generic conditions: it can classify each mode independently and is provided with a synchronized multimodal data set.

## Introduction

Human perception of the environment through multiple senses is what we call multimodality. Human manipulation of objects, a natural example of multimodality, connects the senses of sight and touch from an early age, and this sensory connection is strengthened over the course of child development^1,2^, and continues throughout our lives^3^. In particular, vision and haptics are complementary to each other, improving the credibility of mental representations of object properties and recognition performance^4–7^. In this article, we present an Artificial Cognitive System (ACS) that builds on a multimodality ability using human manipulation data, achieving perceptual awareness and a dynamic capacity to adapt to changing environments.

In the past few years, there has been a growing interest in the development of multimodality in artificial agents^8^, especially robots, as it may facilitate the creation of systems that autonomously adapt to different environments. In particular, thanks to the advances in visuo-tactile sensors for haptic capture systems, researchers are delving deeper into the connection between vision and touch in robotics^9–11^. The tactile patterns that result from these sensors can be related to the images obtained using the visual mode, thus creating a framework that enables the establishment of multimodal relationships. By gathering haptic data in the form of images, the dimensional gap between touch and vision features can be successfully overcome^12^. Furthermore, the maturity of image recognition methods (e.g. deep learning algorithms) has allowed some progress in the study of the relationship between these two modes. However, existing haptic data is still a long way from what would be a haptic dexterous robot exploration similar to that of humans. To solve this problem, researchers have recently designed a glove that can generate tactile patterns through a mechanoreceptor network and also provide information related to the dexterity of the human grasp^13,14^, however, this work does not relate haptic data to visual mode. In this article, we use a haptic capture system that also leverages information from human object manipulation. Synchronized haptic and visual data allow to define and implement a new adaptive and autonomous system in changing environments.

**Figure 1:**
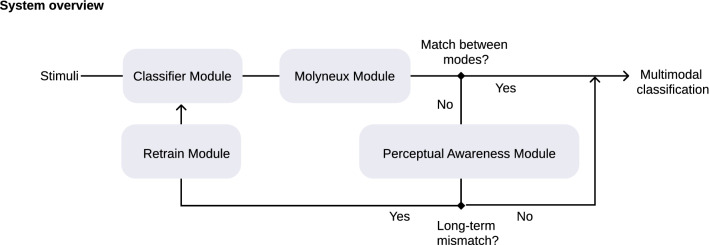
Flow chart of the overall algorithm. Synchronized stimuli (visual and haptic) are classified in the Classifier Module using well-known state-of-the-art algorithms: a CNN classifier for the visual data and a naïve Bayes classifier for the haptic data. Then, the Molyneux Module checks the coherence of the result of the classification of both modes. If the haptic classification and the visual classification match, we say the system is coherent. Otherwise, it must be verified if the incoherence is due to a transient error in a classifier or it persists over time. The Perceptual Awareness Module handles this issue. In the case of a long-term mismatch, the faulty channel is retrained with the relabeled data from the stable channel in the Retrain Module.

To achieve this objective, we designed and printed novel 3D objects that collect human exploration data with multiple capacitive touch sensors on the surface of the objects. With this dataset, our ACS achieves multimodality via transfer learning from touch to vision. Unlike other approaches^11^, where corrupted inputs are incorporated during training time, we present a new mechanism that allows our ACS to use multimodality to continuously monitor whether the information received from visual and haptic modes matches, hence being able to detect anomalies (e.g. blurred vision). Given that the two sensory modalities are independent but collaborative, like those of a human, we examine how our system dynamically changes the strength of the intermodal connection to better solve object recognition problems when we degrade the quality of visual information at test time. If mismatches between vision and touch channels persist over time, the ACS can autonomously retrain the faulty modality through transfer knowledge within few minutes. A flow chart of the overall algorithm is presented in (Fig. 1). Our findings suggest that with the implantation of biologically inspired multimodality, the ACS becomes perceptually aware of a faulty sensory modality and autonomously adapts to changing environments without losing performance.

Although very specific data sets and recognition algorithms have been used in this work, they are not a limitation for the development of our proposal. Moreover, only two conditions on data sets and algorithms are sufficient to implement the proposed mechanisms of perceptual awareness and self-adaptation in changing environments. These are: (i) visual and haptic data sets must be able to be synchronized, (ii) haptic and visual recognition algorithms must be independent. The generic nature of these premises leads to consider the proposed mechanisms as an interesting contribution to the state of the art in real systems.

## Results

### Haptic data and object recognition

**Figure 2:**
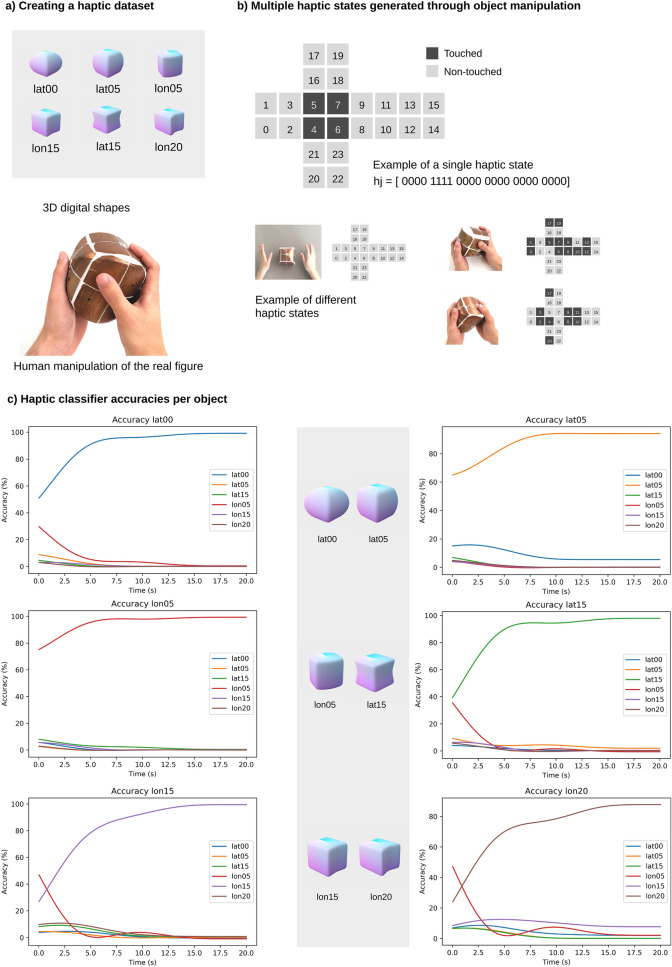
From object human manipulation to automatic haptic classification. (**a**) The objects of this study are six 3D printed shapes labelled as lat00, lat05, lat15, lon05, lon15, and lon20. The participant sits in front of a computer and follows instructions on how to manipulate the objects randomly. (**b**) The human manipulation data of the objects is collected by the system and are stored (forty times per second) as haptic states. A haptic state is represented by a 24-bit array and indicates the status of each sensor (touch/not touched). (**c**) ACS recognizes each object through a simple Bayes algorithm based on haptic states. As show in this subfigure, accuracies higher than 80% are consistently reached after a few seconds.

We designed a system that captures haptic information generated by humans during the object manipulation process. This collected data is enough to create a haptic recognition system that outperforms humans in a classification task with similar 3D shapes^15^, both in accuracy and response time. As illustrated in Fig. 2, the objects are six similar shapes we have digitally created and 3D printed^15,16^. The external surface of each object is completely covered with 24 copper pads that are equally distributed and connected to an electronic board placed inside, which also includes a gyroscope. During the object manipulation, this system samples data from all the sensors at 40 Hz and sends it to a computer through wireless communication. Every sample is stored as a 24-bit array, ${\mathrm{h}}_{\mathrm{j}}$, called haptic state of the $\mathrm{j}$ sample, one bit for each copper pad (Fig. 2b), hence the system has no information about the relationship between the location of the 24 sensors and the positions of their statuses in the array. Besides, since each sensor has its position inside the array, the resulting state would vary if sensors were placed differently. For this reason, and to make sure the presented algorithm does not use the sensors’ order to recognize the objects, sensors are placed in a way that every position in the haptic state corresponds to a sensor located in the same spatial location for each object. In the same way that touch receptors on the human hand would also receive input from corresponding locations on different objects when the relative orientation of the grasping hand and the objects would be held constant.

Our haptic dataset is based on the haptic states described above and their time evolution. The geometry of the objects affects their handling, and this is reflected in our data. To gather our haptic dataset, one participant is invited to manipulate each object with both hands and perform a random exploration task. Four series, each lasting for five minutes, have been recorded per object.

To perform automatic haptic object recognition, the dataset is divided into two parts: three series for training (15 min) and one for testing (5 min). To determine the probability of a set of $n$ consecutive haptic states $({\mathrm{h}}_{1},\dots ,{\mathrm{h}}_{\mathrm{n}})$ belonging to a specific object ${\mathrm{S}}_{\mathrm{i}}$ ($\mathrm{i }\in [1, ..., 6]$), i.e., $\mathrm{P}({\mathrm{S}}_{\mathrm{i}}\mid {\mathrm{h}}_{1},\dots ,{\mathrm{h}}_{\mathrm{n}})$, we adopt a naïve Bayes approach as follows:

$$\widehat{{S}_{i}}= \underset{{S}_{i},\mathit{ }i \in [1, \dots ,6]}{\mathrm{argmax}}\left[P({S}_{i})\prod_{j=1}^{n}P(\left.{h}_{j} \right| {S}_{i})\right]$$

Here the $\mathrm{n}$-product $\mathrm{P}({\mathrm{h}}_{\mathrm{j}}\mid {\mathrm{S}}_{\mathrm{i}})$ is the naïve condition, $\mathrm{P}\left({\mathrm{S}}_{\mathrm{i}}\right)=\frac{1}{6}$ is the probability of each object and $\widehat{{\mathrm{S}}_{\mathrm{i}}}$ the resulting prediction.

Our haptic object recognition system achieves an average accuracy of 89.63% after just 8 s of manipulation (average time for best accuracy in humans^15^), as shown in Fig. 2.

### Multimodal dataset generation and visual object recognition

**Figure 3:**
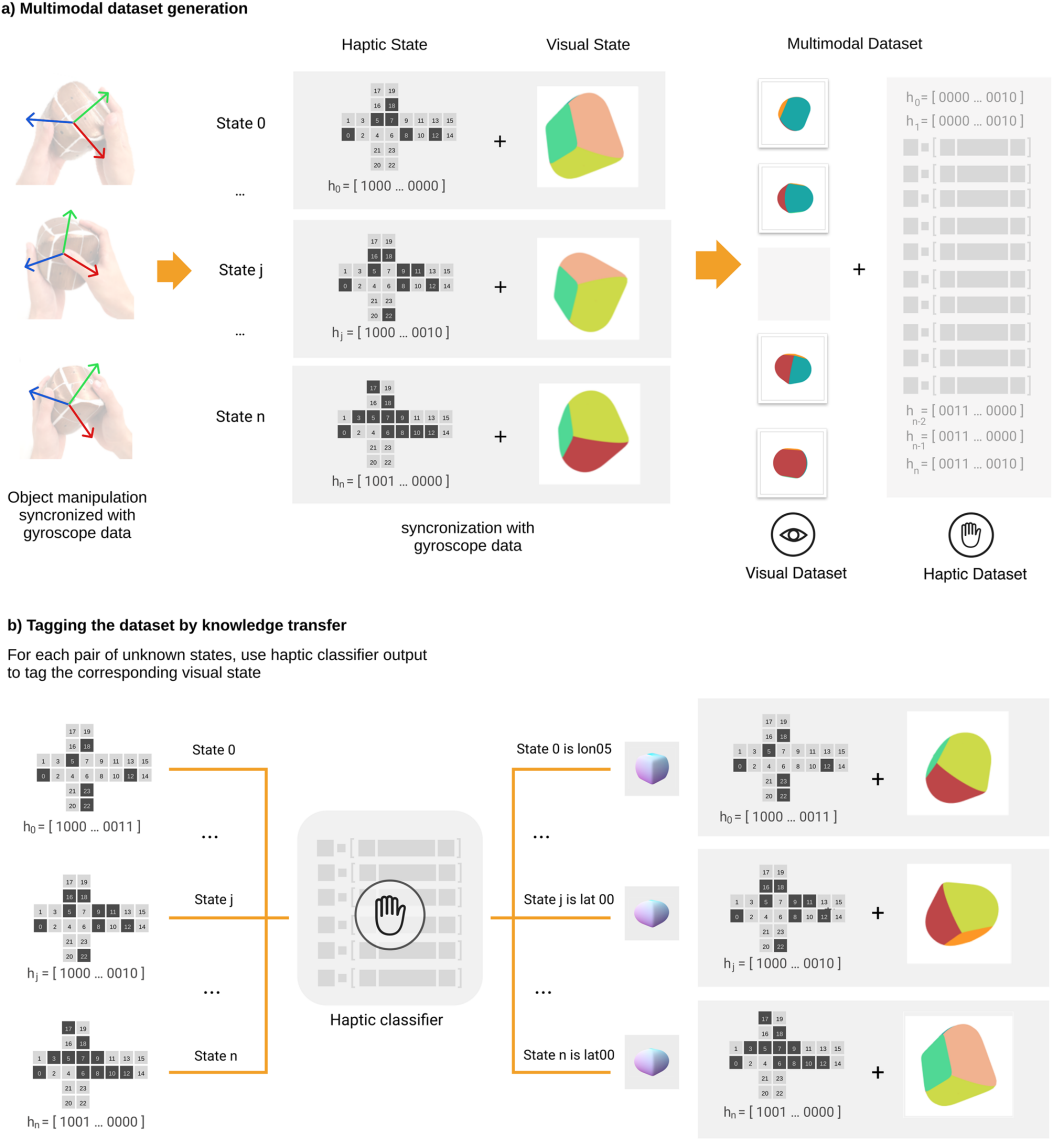
Multimodal dataset generation through knowledge transfer. (**a**) Using the gyroscope, we can obtain the orientation of the object associated to each haptic state. From this information, we can draw (through 3D renders) a visual state of the object and associate it with its corresponding haptic state. From this, given any haptic dataset we can generate its corresponding visual dataset. With this method a new multi-modal dataset is created as a result of the combination of haptic and visual datasets. (**b**) Given that no manual annotation process has been carried out in the creation of the previous multi-modal dataset, the visual mode is not labelled. Here, we propose to tag the visual dataset from the results of the haptic object recognition system. This transfer of information generates a link between the two modes, visual and haptic.

As stated earlier, the 3D printed objects have been produced from a 3D digital render. Using 3D renders and data from gyroscope, we synthesize a video with the movements of the objects caused by human manipulation (Fig. 3). We then create one video frame for each haptic state; see Methods for details. We select the 5-min test data series mentioned above for this purpose. This opens up the visual channel to our system. Now, the ACS can receive data from haptic and visual senses simultaneously (Fig. 3).

**Figure 4:**
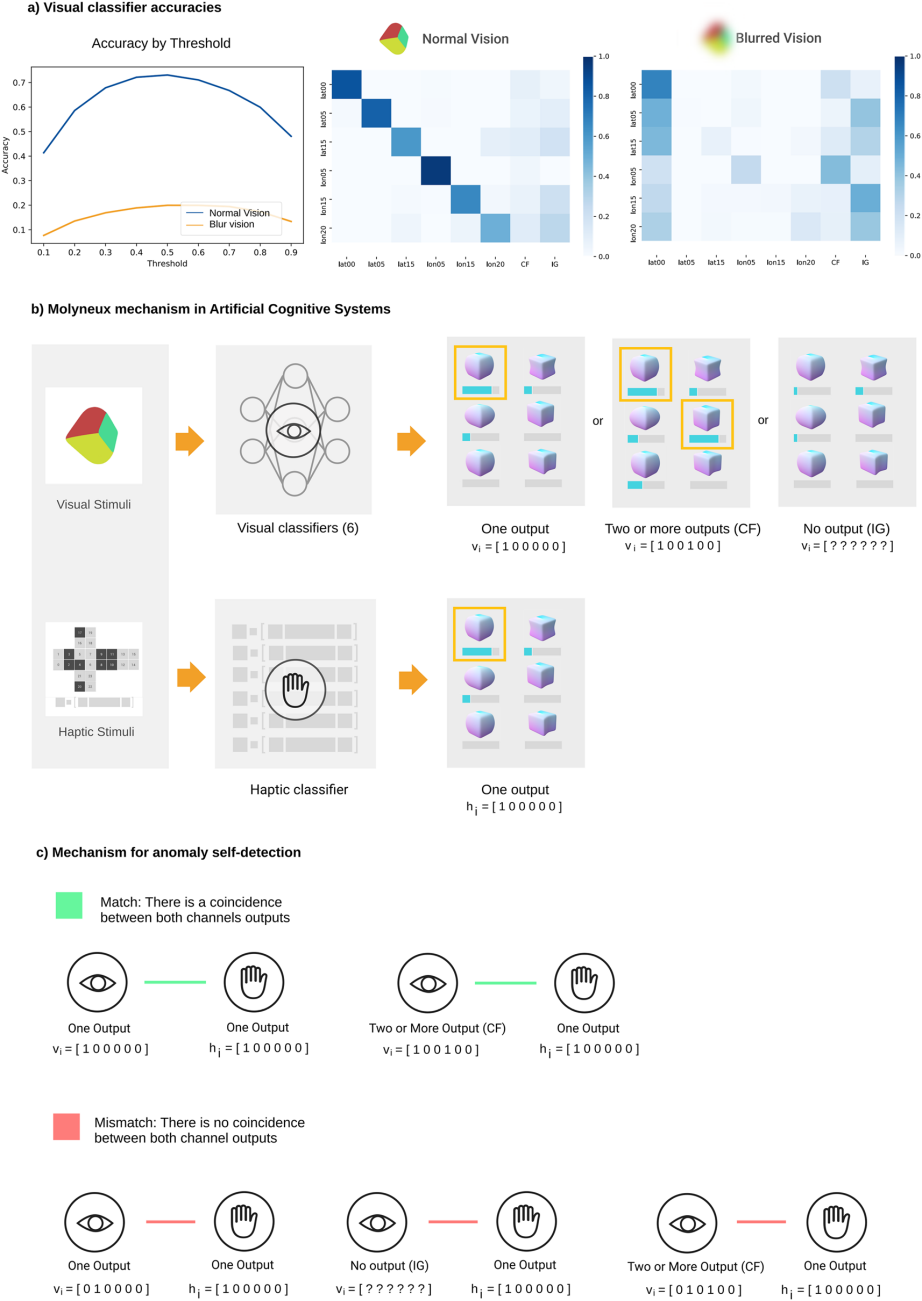
Visual classifier threshold and the Molyneux mechanism. (**a**) With the synthesized video generated from human object manipulation, we trained a one-vs-all based CNN model, generating an individual classifier for each class. In order to determine the output label of a classifier, we have studied which accuracy threshold results in the best performance of the visual model. By setting the threshold to 0.5, we obtain the confusion matrices shown at the bottom after testing the model with normal vision images (like the ones used for training) and blurred vision images. (**b**) Classifier Module. Every video frame and its corresponding haptic state are classified by the visual and haptic object recognition systems, respectively. There are three possible outputs of the visual recognition system: (i) a single class, (ii) multiple classes (CF), or (iii) none (IG). On the other hand, the label from the haptic recognition system is always univocal. **(c)** Molyneux Module. By applying the Molyneux mechanism, we check if visual and haptic recognition agree, which we call a match. Otherwise, we call this disagreement a mismatch. In the case where visual recognition result is CF, it will be a match if one of the possible classes agrees with the haptic classification. All mismatch situations are shown.

Since the ACS has previously had a haptic experience, it can autonomously tag the visual dataset through the results of the already trained haptic object recognition system, creating a multimodal relationship. Obviously, from this transfer of knowledge, we can train a visual object recognition system from the new labelled visual dataset. We divide the 5-min visual dataset into three parts: 60% for training, 20% for testing, and reserve the remaining 20% for a later experiment. Using this data, we train a set of CNNs for visual object recognition. The visual object recognition system is based on a one-vs-all strategy combining six CNNs classifiers, thus yielding a six-dimensional output, $\mathrm{v}\in {\mathbb{R}}^{6}$. Each component of the output, ${\mathrm{v}}_{\mathrm{i}}$, is associated to the probability that the visual input corresponds to one of the 6 stimuli ${\mathrm{S}}_{\mathrm{i}}$ (objects). To decide which is the corresponding object, the following criterion is adopted: a sample belongs to a certain class if only one of the values ${\mathrm{v}}_{\mathrm{i}}$ exceeds a threshold, $\uptau$, ($\exists {!\mathrm{v}}_{\mathrm{i}}$ s.t. ${\mathrm{v}}_{\mathrm{i }}>\uptau$). The value of $\uptau$ is obtained from the relationship between the accuracy of the visual classifier and the different threshold values as shown in Fig. 4. Two other results can be obtained: a) Confusion (CF): there is more than one ${\mathrm{v}}_{\mathrm{i}}$ value above the threshold ($\exists {\mathrm{v}}_{\mathrm{i}},{\mathrm{v}}_{\mathrm{j}}$ s.t. ${\mathrm{v}}_{\mathrm{i }}>\uptau$ and ${\mathrm{v}}_{\mathrm{j }}>\uptau$ with $\mathrm{i}\ne \mathrm{j})$, that is, the visual object recognition system assigns the sample to more than one class. b) Ignorance (IG): there is no value of ${\mathrm{v}}_{\mathrm{i}}$ above the threshold (${\mathrm{v}}_{\mathrm{i }}<\uptau , \forall \mathrm{i}$), i.e., there is a lack of knowledge to decide which class it belongs to. Although there are several efficient methods for dealing with confusion, especially based on heuristics, at this stage we have preferred to include it as a false negative, even at the expense of visual object recognition performance. We will resolve the confusion with the help of the haptic mode, as we will see in the next section. Visual object recognition results shown in Fig. 4a demonstrate that the visual channel of our ACS achieves 75% accuracy including CF and IG as false negatives.

### Stressing the visual channel and the Molyneux mechanism

The Molyneux problem addresses the following question: would a person born blind that later regains sight as an adult, be able to visually recognize the shapes of objects previously experienced by touch?^17^ Recent empirical studies have pointed out that upon recovery of sight, subjects are initially unable to recognize these objects visually. However, after they experience the world with both senses, in a few days a link is created allowing them to pass the Molyneux test^18^. In the present study, this connection between the two senses equips the ACS with a cognitive mechanism that allows it to autonomously detect a faulty channel.

The aforementioned mechanism, which we have called the Molyneux mechanism, allows the ACS to continuously check if what it is seeing coincides with what it is touching. Hence, the ACS can detect sudden anomalies by comparing the classification labels of the two independent channels, haptic and visual (Fig. 4c).

More specifically, the ACS can encounter four different situations while comparing the labels given by the haptic and visual object recognition systems: (i) both recognition systems agree, i.e., there is a match, (ii) do not agree, i.e., there is a mismatch, (iii) the visual object recognition system does not have enough knowledge to classify that sample (IG), which results in a mismatch, and (iv) the visual channel object recognition system assigns the sample to more than one class (CF) and the ACS checks if one of this classes agrees with the haptic recognition (match) or, on the contrary, it does not (mismatch). It is worth noting that when both recognition systems are working properly, some short duration mismatches can occur, but the most common situation is a continuous agreement between the visual and the haptic recognition systems. As these mismatches are short-lived, they can be easily removed with a low pass filter; see Methods for details.

**Figure 5:**
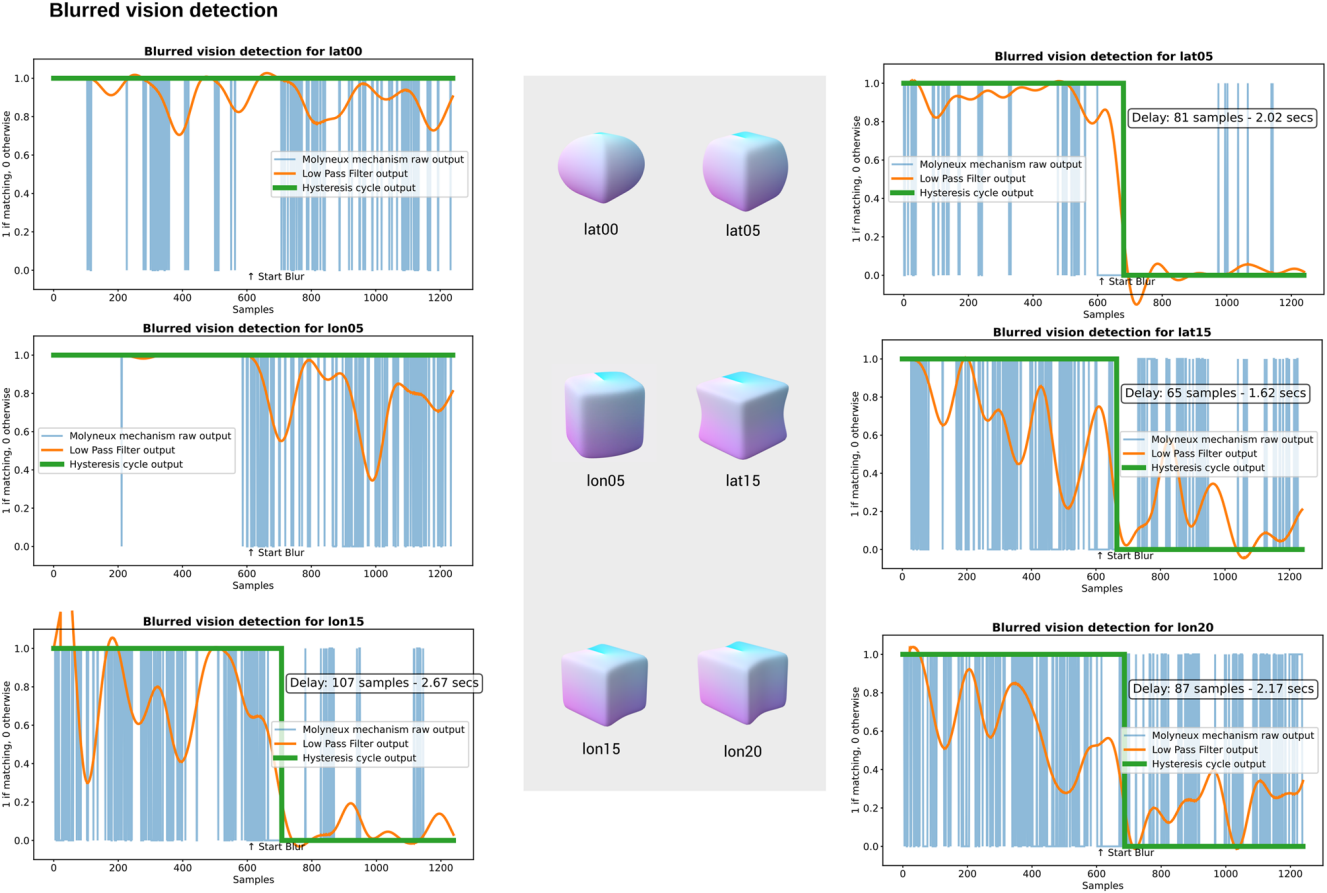
Blurred vision detection through Molyneux mechanism and filtering. By applying the Molyneux mechanism to every visual-haptic pair, the ACS can determine if the two channels are matching or not, hence it can detect failures in one channel. Although there is a clear trend towards the two channels matching when both are working properly, short mismatches can appear (blue plot). In order to obtain an accurate decision whether the channels are matching or not, a low pass filter is applied to attenuate these mismatches (orange plot). Finally, as a way to offer a binary response that decides if the vision is blurred or not, the filter output goes through a hysteresis cycle (green plot). With this entire process the detection of the blurred vision is not immediate and has an average delay of 2.13 s (85.33 samples). Note that there is no mismatch in lat00 and lon05, that is, for these two objects the blur filter does not affect the recognition system.

In order to test this mechanism and study the effectiveness of artificial multimodality for perceptual awareness, we stressed our visual channel by applying a blur filter. Once applied, the accuracy of the visual object recognition system went down to approximately 20% for the 6 classes (see Fig. 4a). The ACS detected this anomaly using the Molyneux mechanism, and did so quickly, with an average delay of only 2.13 s (85.33 samples) after applying the blur filter to the visual input. In Fig. 5 we show the results for each object.

### Self-adaptation to a new environment

**Figure 6:**
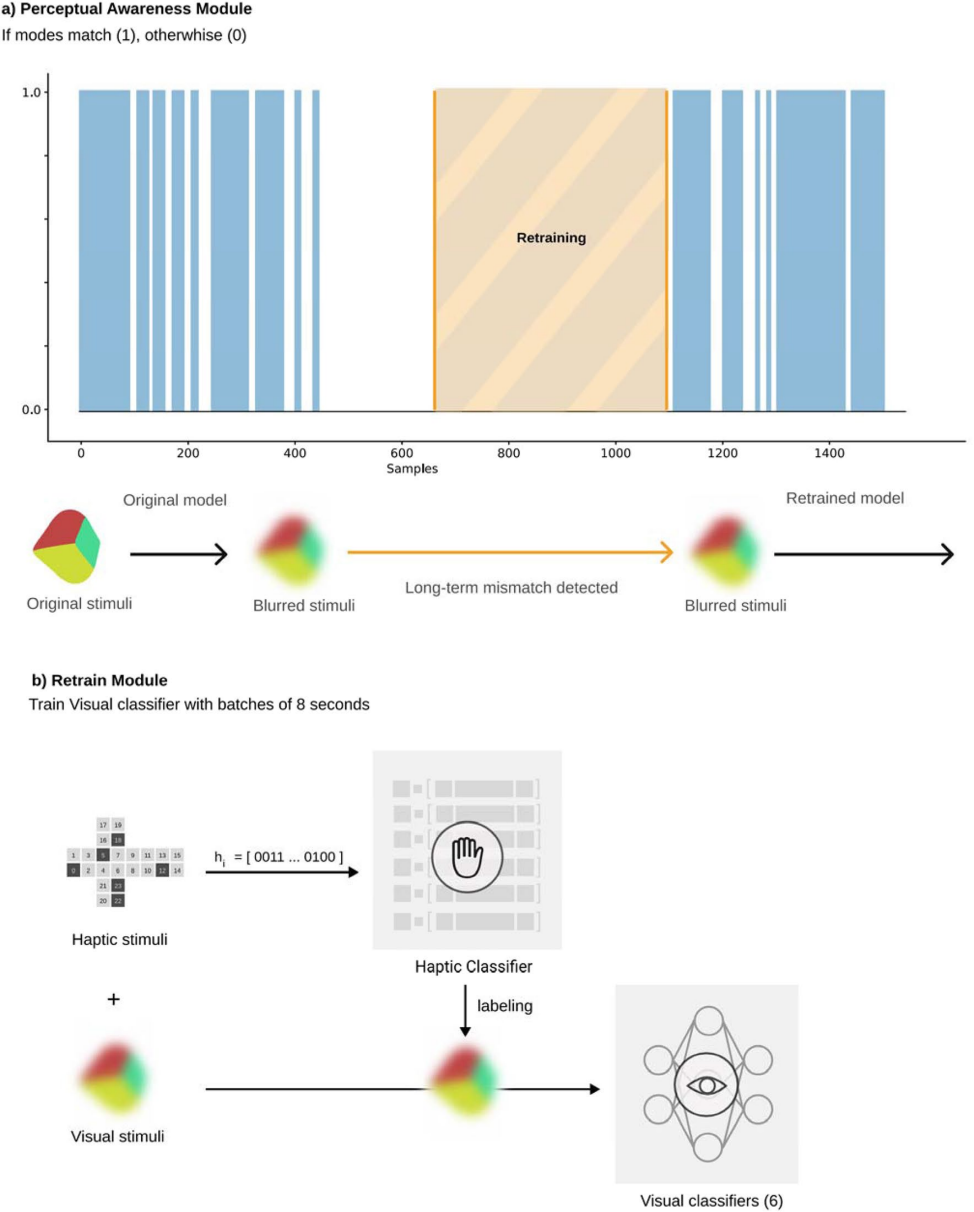
Perceptual awareness and retraining modules. (**a**) The Perceptual Awareness Module is responsible for checking whether the classification incoherence of the modes is short or long-term. If a long-term mismatch is detected, then the retraining module is activated. (**b)** Since the visual stimulus is now blurred, the visual classifier becomes unstable. On the other hand, haptic recognition remains stable as this mode is not affected. Blurred visual stimuli are relabeled with haptic data and used to retrain the visual classifier.

**Figure 7:**
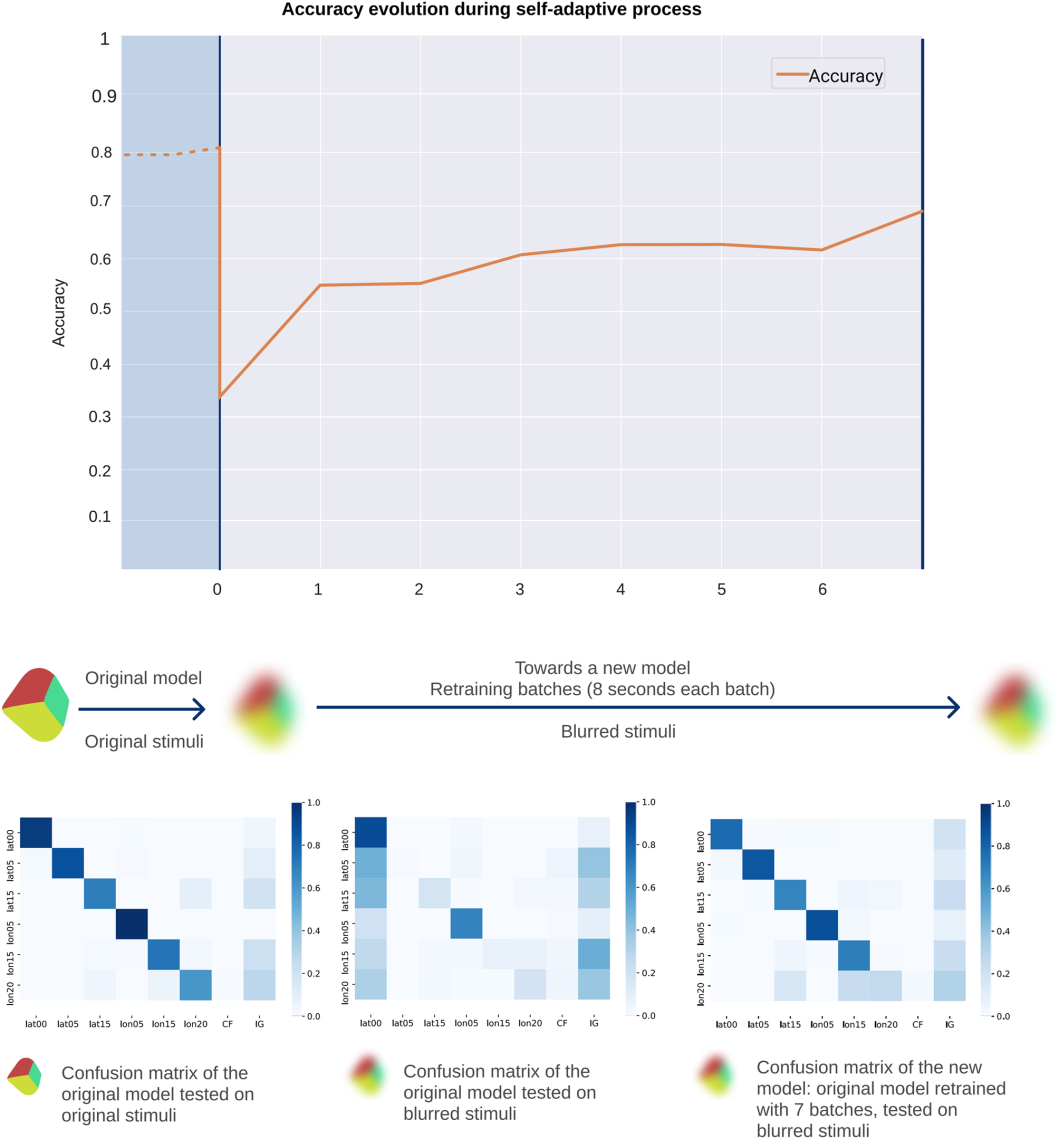
Visual model adaptation to new stimuli acquisition conditions. Top plot shows the decrease in accuracy at the moment (t = 0) when blurred stimuli are first introduced. As blurred batches are incorporated to retrain the system, previous accuracy with original stimuli is now achieved with blurred stimuli (t = 7). Bottom plots show the confusion matrices at the most relevant moments during this adaptation process: (Left) Original model tested with original stimuli, (Center) Original model tested with blurred stimuli (t = 0), (Right) New model after the adaptation process with 7 batches of blurred images (t = 7) tested with blurred stimuli.

The Molyneux mechanism enables ACS to realize, in a fully automatic way, that the haptic and visual object recognition systems lose coherence when a blur filter is applied to the visual channel. This situation does not affect the haptic classifier, which remains stable and consistently provides the visual channel with trustworthy information. Thus, the blurred images are tagged with the output of the haptic classifier, and the ACS can retrain the CNNs of the visual object recognition system in order to classify them correctly (The modules in charge of this process are described in Fig. 6). Using the 20% (60 s) of the visual dataset that we had previously reserved after going to a blur filter, we retrained the visual object recognition system by grouping the retraining samples into 8-s batches (total of 7 batches) and iteratively feeding the CNNs with one batch at a time. For each retraining batch iteration, we calculate the current accuracy of the visual object recognition system in the blurred vision scenario. As shown in Fig. 7, the accuracy in the blurred vision scenario increases as the ACS receives more blurred visual information in its retraining process.

## Discussion

### Visual object recognition and multimodality

As described in the Results section, the visual mode benefits from the previous experience of the haptic mode. The haptic classifier recognizes the object being manipulated and the ACS tags the information of the visual channel in real-time. This would be equivalent to the process when a human becomes aware of the connection that exists between a known manipulated object and its visualization for the first time. It is important to highlight the autonomy that this knowledge transfer gives the ACS, since it does not need an external agent to tag the visual channel (Fig. 3b).

On the other hand, heuristic arguments are commonly used to solve the confusion in the outputs of one-vs-all visual classifiers. However, in our approach, multimodality allows us to solve confusion through the haptic mode, i.e. through the interaction with the environment (object). In this study, if we use a highest value heuristic, our visual model reaches an accuracy of 79%, whereas if we use multimodality to solve confusion, the accuracy goes up to 81%. Although in this case accuracies are similar, if the haptic classifier is stable, the multimodality will always equal or improve the results of heuristic methods. This result resembles the exchange of multi-modal information to discriminate a stimulus in humans, which is a very common process^4^.

### Perceptual awareness

The Molyneux mechanism defined in this article allows the ACS to check the coherence between two synchronized samples (haptic-visual) (Fig. 4c). In other words, this mechanism allows the ACS to answer the question: is what I touch and what I see the same object? As shown in Fig. 5, in the Results and also in Methods section, the study of short periods of incoherencies has been detailed to differentiate them from those that are longer in duration. Filtering visual-haptic classification pairs in real-time allows the ACS to realize that the visual object recognition system is not working properly when the blur filter is applied. High and stable accuracy over time of the haptic classifier is assumed for this study as shown in Fig. 2. The goal of the filtering process is to identify changes in the visual channel. It can be observed (see Fig. 5) that for objects lat00 and lon05 the change to blurred vision is not detected. This is because the visual object recognition system continues to classify these two objects correctly despite the blur, and therefore there is no incoherence of any kind other than a decrease in visual accuracies for these two objects, i.e., the filter does not detect any change in the visual channel.

### Resilience from multimodality

Realizing that the environment has changed is the first step in the process of self-adapting to it. The proposed design with two independent object recognition systems and the multimodality approach allows the ACS to autonomously adapt to changes in the environment that affect one of its sensory modes using the information of the mode that remains stable. The results from Fig. 7 show how after just one minute of retraining the visual classifier (CNN), the ACS adapts to the blurred scenario with an accuracy of 69.1%.

As described, the perceptual awareness mechanism is based on checking for coherence in the classification of synchronized pairs of visual and haptic data. There are no conditions on the type of classifiers or their accuracy. However, it should be noted that the fewer samples needed to recognize the stimuli, the shorter the periods of mismatches in the perceptual module and the easier it will be to detect long-term mismatches. On the other hand, the fact that recognition algorithms are independent allows us to re-label one mode through the other, a key step in retraining the failed mode. Finally, the transfer of knowledge between modes has in this work a relevant ecological character in the sense proposed in (18), since ACSs could adapt to transformations or changes in their perception systems during their lifespan.

## Limitations

One of the main limitations of this work is its extension to other different settings as it would be necessary to sensor the new objects in order to capture data from haptic interactions. This capture system has been used to emulate a possible (and not yet existing) artificial system that manipulates objects as a human does. Despite its limitations, the interest of this capture system lies in its high sampling frequency that allows us to retrain the visual recognizer in just one minute. This feature seems relevant and perhaps should be taken into account in the design of future haptic capture systems.

With the approach proposed in this article, we are aware that we are simplifying the problem by using synthetic images and avoiding the occlusions caused by human hands during object manipulation. Nonetheless, even though this work is not focused on solving this issue, this apparent problem could be part of the solution, since these occlusions are strongly correlated with haptic data.

Another limitation of this approach is that, in this very first experiment where we have shown the benefits of multimodality for self-adaptation in a changing environment in which the vision channel suddenly becomes blurred, we have not accounted for other errors that may cause the vision channel to stop working properly. Moreover, we have used single frames instead of a group of states in the visual model. Our goal with the presented experiment was to test the Molyneux mechanism and show the benefits of multimodality for environment self-adaptation for object recognition tasks. We believe this is the very first step towards the development of perceptual awareness in ACSs for adaptation to changes in their perception systems, and further studies are required.

## Opportunities for future research

Although the current trend is to place touch sensors on robotic hands’ end-effectors, the use of sensors on objects is an equally important field of research, especially to obtain data for ACS. In fact, it seems reasonable to assume there would be a correlation between what was obtained by the introduced haptic capture system and the point cloud that a robotic hand could generate if it could interact with that system. We hypothesize that placing sensors directly on objects is equivalent to obtaining data from a human-like robotic dexterous hands. This would allow us to integrate our ACS into a robotic hand such as the Shadow Hand^19^. Other researchers have showed that this hand^20^ could exhibit high levels of dexterity in object manipulation tasks.

It would also be interesting to study other ways to stress the input channels. Two situations of special interest are: (a) the inducement of errors in the haptic channel to study multimodality in the opposite direction, (b) the desynchronization between the haptic and visual channels.

Finally, to extend this work to crossmodality, a deeper study of the Molyneux mechanism is necessary. To do this, we propose two approaches. On the one hand, we could create an integrated sensory model where orientation-specific visual inputs are associated with orientation-specific haptic inputs. It is possible that this model will produce more refined representations and better results. On the other hand, we propose an analysis of the haptic and visual perceptual spaces used in neuroscience^21,22^, which would allow us to understand how we can relate unknown objects with a previous ACS experience. These perceptual spaces could work as an artificial internal representation, perhaps a first step in reproducing the human mental simulation of physical actions, a key point for the elaboration of abstract concepts as evidenced by the results of cognitive psychology^23–25^.

## Methods

In this section, we provide the methods and procedures used in this research article.

### Visual dataset generation

To generate the 3D renders, the six similar shapes meshes are placed independently on a Processing 3D scene with a 299 × 299 window to ensure the generation of square images during the rendering process. To this end, we use a dataset consisting of four files per object (20 min in total). Then, an independent rendering process has been performed for each of these files to match our haptic dataset. In each scene, the object of study is initially positioned in the world origin (0,0,0), which is the central point of view of a camera that remains static during the whole process. This camera position is the same for all the experiments.

Once the environment is set up, each object is texturized uniformly generating a UV Map with the same colors on each side of the shape. Using this approach, each side can be easily identified despite the symmetries present in all the shapes. Then, the data from the gyroscope collected during objects’ human manipulation is used to perform rotations on the object that match those from the human manipulation experiment. Each haptic state associates four values to each component of a quaternion in order to perform each rotation, considering the center of the figure the origin of the rotation. This approach recreates the objects´ original movements since the gyroscope in the 3D printed shapes is placed inside the center of the object.

This rotation is performed for each haptic state and rendered from the world camera generating images that capture each current object rotation forming the visual dataset. This synchronization between the visual and the haptic dataset is what makes the experiments presented in this article possible.

### Blurred images generation

In order to simulate a sudden loss of visual channel, we generate blurred images for all samples. These blurred images are generated by applying an average filter over the original images, convoluting the image with a 20 × 20 normalized box filter from OpenCV libraries.

### Visual object recognition system and training process

In order to generate the visual recognition system, we follow a one-vs-all strategy. This strategy generates an independent visual classifier for each one of the six classes (${\mathrm{Model}}_{[\mathrm{c}]}$ where c defines the class). ${\mathrm{Model}}_{[\mathrm{c}]}$ classifies a single image as belonging to class c or not. We adapt a pre-trained CNN model based on InceptionV3 architecture^26^, and we change the last layer for a fully connected dense layer with 2 outputs, using a softmax activation function. Each of the ${\mathrm{Model}}_{[\mathrm{c}]}$ classifiers is trained using categorical cross-entropy as loss function and a dropout of 0.4. The training is performed for 5 epochs using 60/20 split of our own dataset for training/test the models (the remaining 20 percent is reserved for the blur filter test). Samples of our dataset are split into two classes for each of the Model[c]: (i) positive class: samples belonging to class c, and (ii) negative class: samples that not belong to class c. Pre-trained initial models are initialized using the weights of ImageNet dataset^27^.

### Visual retraining process

By using the remaining 20% of samples that were not used in the model training/testing process (which amount to around 60 s), each of the ${\mathrm{Model}}_{[\mathrm{c}]}$ classifiers is retrained using exactly the same parameters used for training the original ${\mathrm{Model}}_{[\mathrm{c}]}$. The difference now is that the initial CNN weights are those obtained after the previous training process. This retraining process is performed using one batch (8 s of samples) at a time, and is repeated sequentially up to 7 times, as the samples are grouped in 7 batches. This retraining process simulates the gradual adaptation of the visual model to new conditions, that in this work we simulate by a sudden loss of vision resulting in an input of blurred images. By using the presented gradual retraining process, the model adapts to these new visual conditions for the blurred vision scenario.

### Molyneux mechanism and filtering

The haptic and visual classifiers constantly classify the haptic states and visual frames of the figures that are acquired at a 40 Hz frequency. The Molyneux mechanism enables us to compare the haptic classification with the visual classification every 25 ms, checking if (i) both channels are in agreement, and (ii) there is a failure in one of the channels or not.

It is normal that some short duration mismatches between both channels appear even if both channels are working properly. In order to provide a stable decision regardless of whether a channel is failing or not, a low pass filter is applied to the output of the Molyneux mechanism.

The low pass filter consists in a 6th order Butterworth filter offering a flat output for the passband frequencies and avoiding ripples. The first 1000 samples of each figure test file are used to study the duration of the mismatches when there is no failure in either of the two channels, obtaining a mean duration of *µ* = 3.3 samples and a standard deviation of *σ* = 9.1. Since our aim is to achieve a huge attenuation for the mismatches frequencies, the cutoff frequency is set a decade before the frequency corresponding to the *μ* + *σ* duration. Taking into account the sample rate of 40 Hz, the cutoff frequency can be calculated as:

$${f}_{cutoff}=\frac{{f}_{s}}{10 \cdot \left(\upmu +\upsigma \right)}=0.32 Hz$$

After applying the filter, the output of the Molyneux mechanism fluctuates between 0 and 1 (see the orange plot in Fig. 5), where 0 corresponds to channels that do not match (failure in one channel) and 1 to channels that match. In order to offer a binary response, such as the one show in Fig. 5, the filter output goes through a hysteresis cycle, where the output goes from 0 to 1 if the input is higher than 0.8 and from 1 to 0 if the input is lower than 0.2.

## Ethics declarations

All methods were carried out in accordance with relevant guidelines and regulations. Informed consent was obtained from the tester prior to manual manipulation of 3D printed objects.
